# The Antibacterial Activity of Crude Extracts of Secondary Metabolites from Bacterial Endophytes Associated with *Dicoma anomala*

**DOI:** 10.1155/2021/8812043

**Published:** 2021-04-12

**Authors:** Sephokoane Cindy Makuwa, Mahloro Hope Serepa-Dlamini

**Affiliations:** Department of Biotechnology and Food Technology, Faculty of Science, University of Johannesburg, Doornfontein Campus, Johannesburg, South Africa

## Abstract

Endophytic bacteria isolated from medicinal plants are recognized valuable sources of novel bioactive compounds with various activities such as antimicrobial, anticancer, and antiviral. In this study, eleven bacterial endophytes were isolated from surface sterilized roots and leave tissues, of medicinal plant *Dicoma anomala*. The bacterial endophytes were identified by sequencing the 16S rRNA gene, and belong to five genera *viz Bacillus, Staphylococcus, Stenotrophomonas, Enterobacter*, and *Pantoea*. The dominant genera were *Bacillus* with five strains, *Staphylococcus* with two strains, and *Stenotrophomonas* with two strains. The crude extracts of seven selected bacterial endophytes indicated antimicrobial activity against five pathogenic strains *Escherichia coli* (ATCC 25922), *Bacillus cereus* (ATCC 10876), *Staphylococcus aureus* (NCTC 6571), *Pseudomonas aeruginosa* (ATCC 27853), and *Klebsiella oxytoca* (ATCC 13182), with significant inhibition concentration ranging from 0.312 mg/ml to 0.625 mg/ml. Finally, based on the data analysis of the crude extracts of the endophytes, we identified bioactive secondary metabolites with reported biological activities such as antimicrobial, anti-inflammatory, and antioxidant properties with biotechnological applications in medicine, agriculture, and other industries. This study reported for the first time bacterial endophytes associated with *D. anomala,* with antimicrobial activity against bacterial pathogens.

## 1. Introduction

Medicinal plants are a source of biologically active compounds for novel drug development [1]. Historically, plants with healing properties were utilized in continents such as Asia and Africa, to treat diseases such as diarrhoea, headaches, and fevers [1]. In modern days, approximately 80% of the world's population, especially within the developing countries, rely on herbal medicines from traditional healers to treat various diseases [2].

The use of medicinal plants for novel drug discovery is however a limiting factor, because at times high amounts of the plant materials are required for clinical studies before the products even make it to the market; moreover, some compounds are derived from endangered or endemic plant species. One of the advances made in addressing these concerns was the discovery of microorganisms called endophytes living within plants, which produce the same or similar compounds like those produced by their plant hosts [1, 3]. Endophytes are either fungi or bacteria and reside inside the plant tissues without causing any harm. Of interest in the current study are bacterial endophytes. Endophytic bacteria belong to various genera and many of the biological active substances extracted from endophytic bacteria are reported to be novel with various bioactivities [4]. Microbes are a good source for extraction of biologically active compounds due to their ease of isolation, growth, and inability to impact negatively on the environment and agricultural productivity [5, 6]. From a commercial and industrial point of view, it is easier to expand microbial fermentation which can ultimately increase production of biologically active compounds. Thus, microbes associated with medicinal plants present an opportunity for isolating novel biologically active compounds, with benefit of preserving medicinal plants, as minimal plant material is required for endophyte isolation.

Endophytes significantly ameliorate plant growth by a number of mechanisms including increase of uptake of essential nutrients such as phosphate, ammonia, and nitrogen, producing phytohormones such as indole-3-acetic acid and gibberellin, and inhibiting pathogen proliferation through the production of secondary metabolites with antimicrobial activity [1]. Moreover, many antimicrobial compounds produced by bacterial endophytes belong to several structural classes such as peptides, alkaloids, steroids, quinines, terpenoids, phenols, and flavonoids with various applications [7]. Therefore, there is a worldwide renewed scientific effort to isolate endophytes and study their natural products, which play a vital role as antibacterial, antiviral, antioxidant, antiarthritic, and antidiabetic and as immune-suppressive compound.

*Dicoma anomala* is a perennial herb with an underground tuber; it belongs to the family Asteraceae. *Dicoma anomala* is mostly distributed in southern African countries; in South Africa it is found in Limpopo, North-West, Gauteng, Mpumalanga, Free State, Northern Cape, and KwaZulu-Natal provinces [8, 9]. *Dicoma anomala* roots and leaves have ethnomedicinal uses for ailments such as colds and coughs, fevers, ulcers, labour pains, dysentery, stomach problems, dermatosis, sores, and wounds [8]. Literature indicates that *D. anomala* is known to produce secondary metabolites including acetylenic compounds, diterpene, flavonoids, phenolic acids, phytosterols, saponins, sesquiterpene lactones, tannins, and triterpenes [10] and a study reported by [8] has shown that these compounds possess antimicrobial, anti-inflammatory, antioxidant, antiplasmodial, anticancer, and cytotoxicity, antihyperglycaemic, and hepatoprotective properties. Consequently, extracting and identifying novel secondary bioactive compounds from bacterial endophytes associated with this plant could become the alternative option to overcome levels of drug resistance and conserve the plant species eventually.

This study was undertaken to isolate and identify bacterial endophytes from *D. anomala*, which is a less explored medicinal plant with ethno-botanical history. In addition, the selected endophyte's secondary metabolite crude extracts were assayed for antibacterial activity and further identification of the secreted secondary metabolites using Gas Chromatography High-Resolution Time-of-Flight Mass Spectrometry (GC-HRTOF-MS). To the best of our knowledge, there has been no previous work published on antimicrobial activity of bacterial endophytes isolated from medicinal plant *D. anomala*.

## 2. Materials and Methods

### 2.1. Plant Sample Collection and Identification

*Dicoma anomala* plant material was collected in April 2018 from the natural habitat with slit soil type in Eisleben, Limpopo province, South Africa (23.52 S 29.824 E). Whole plants including roots were placed in sterile polyethylene bags and transported to the laboratory under 4°C. The identification of the plant material was carried out at the University of Johannesburg Herbarium (JRAU) and a sample specimen of the plant material was deposited JRAU with voucher specimen number Serepa-Dlamini 204 and species name *Dicoma anomala*. The remaining collected plant material was immediately processed in the laboratory.

### 2.2. Isolation of Endophytic Bacteria

Immediately after collection, in the lab, endophytic bacteria were isolated from fresh leaves and roots of the plant following the procedure described by [11]. In brief, plant parts were washed thoroughly with running tap water and surface sterilized with 70% ethanol for 5 minutes, followed by a rinse with sterile distilled water, soaking in 2% NaClO for 3 minutes, and final rinse with sterile distilled water 3 times and the final wash water was plated on to nutrient agar plates as control. The surface-disinfected plant parts were crushed using sterile mortar and pestle and macerated with phosphate buffer (8 g NaCl, 0.2 g KCl, 1.44 g Na_2_HPO_4_, and KH_2_PO_4_ at pH 7.4). Following sterile techniques, the homogenate was streaked on nutrient agar plates and incubated together with the control plates at 28°C for 2–7 days. The plates were observed for growth daily; grown colonies were subcultured several times until pure single colonies were obtained. Confirmation of culture purity of the strains was selected on the basis of phenotypic characteristics such as colony morphology, colony colour, colony size, and Gram reaction. In total, 11 isolates were selected for this study and preserved in 30% glycerol stock (v/v) solution for long-term storage at −80°C.

### 2.3. Genotypic Characterization of the Bacterial Endophytes

#### 2.3.1. Genomic DNA Extraction

Genomic DNA was extracted from pure solid colonies using the Zymo Research Fungal/Bacterial DNA MiniPrep Kit as per manufacturer's protocol (Zymo Research, USA). The extracted DNA was electrophoresed on 1% agarose gel and the concentration was determined using the Nanodrop Spectrophotometer (Thermo Fisher Scientific, USA).

#### 2.3.2. 16S rRNA Gene Sequence Analysis

Using Polymerase chain reaction (PCR), DNA of each bacterial isolate was amplified using 16S rRNA gene universal primers described by [12]. The amplification of the 16S rDNA was carried out in a reaction tube with a final volume of 25 *μ*L containing 2.5 *μ*L (<1,000 ng) of template DNA, 2.5 *μ*L (10 *μ*M) of each forward primer 5′-AGAGTTTGATCCTGGCTCAG-3′F and reverse primer 5′-AAGGAGGTGATCCAAGCCGCA-3′ R, 4 *μ*L of 2X PCR Master mix with standard buffer (20 mM Tris-HCI, 1.8 mM MgCl_2_, 22 mM NH_4_Cl, 22 mM KCl, 0.2 mM dNTPs, 5% glycerol, 0.06% IGEPAL® CA-630, 0.05% Tween® 20, 25 units/mL One Taq® DNA polymerase) and final volume was filled up 25 *μ*L with nuclease-free water. A negative control (PCR mix without DNA) was included in all PCR experiments. Using MyCycler™ Thermal Cycler (Bio-Rad, USA), the PCR reaction conditions were as follows: initial denaturation at 94°C for 3 minutes, followed by 35 cycles of denaturation at 94°C for 1 minute, annealing at 48°C for 1 minute and extension at 72°C for 2 minutes, and a final extension of 72°C for 10 minutes. The PCR products were purified with ExoSAP-it™ (Thermo Fisher Scientific, USA) and sent for sequencing at a commercial service provider Inqaba Biotechnical Industries (Pty) Ltd, Pretoria, South Africa.

#### 2.3.3. Phylogenetic Analysis

Raw sequence data were used to create consensus sequences using BioEdit Sequence Alignment Editor version 7.2.6 [13]. The obtained 16S rDNA sequences were subjected to Basic Local Alignment Search Tool (BLAST) analysis against the rRNA sequence database (Bacteria and Archaea) at the National Centre for Biotechnology Information (NCBI) (available at http://blast.ncbi.nlm.nih.gov) to compare with the closest related bacterial species. Only bacterial species with highest similarity identity percentage (90–100%) were selected for phylogenetic analysis. Phylogenetic trees were constructed using MEGA 7 [14] after alignment with MUSCLE [15]. DNA substitutions were done according to the Tamura Nei model. Phylogenetic relatedness of the endophytes with other close relatives was clustered using maximum-likelihood method. Bootstrap replications of 1000 were used as the statistical confidence of the nodes in the phylogenetic trees. Outgroups were considered to support differences among species. The nucleotide sequences of 16S rRNA genes were deposited to GenBank ((https://www.ncbi.nlm.nih.gov/genbank/) and assigned accession numbers.

#### 2.3.4. Nucleotide Sequence Accession Numbers

The bacterial isolates were assigned the following accession numbers: MN029049 (*Stenotrophomonas* sp. strain MHSD20), MN029050 (*Enterobacter* sp. strain MHSD22), MN029051 (*Staphylococcus* sp. strain MHSD24), MN029052 (*Staphylococcus* sp. strain MHSD26), MN029053 (*Bacillus* sp. strain MHSD28), MN078165 *Bacillus* sp. strain MHSD13, MN078166 (*Bacillus* sp. strain MHSD14), MN078167 (*Bacillus* sp. strain MHSD16), MN078168 (*Bacillus* sp. strain MHSD17), and MN093331 (*Pantoea* sp. strain MHSD15).

### 2.4. Antimicrobial Assay Minimum Inhibitory Concentration (MIC)

#### 2.4.1. Preparation of Bacterial Endophyte's Secondary Metabolite Crude Extracts

Secondary metabolites were extracted from selected isolated bacterial endophytes; care was taken to include species which were not assayed previously. The selected species included *Stenotrophomonas* sp. strain MHSD20, *Enterobacter* sp. strain MHSD22, *Staphylococcus* sp. strain MHSD26, *Bacillus* sp. strain MHSD28, *Stenotrophomonas* sp. strain MHSD12, *Bacillus* sp. strain MHSD14, and *Pantoea s*p. strain MHSD15. The bacterial endophytes were cultured in 1L Luria Broth (LB) for 7 days at 28°C on a shaking incubator at 180 rpm. After cultivation, 2 g/L of sterilized Amberlite® XAD-7-HP resin (Sigma-Aldrich, Darmstadt, Germany) was added to the cultures and shaken for 2 hours at 180 rpm, to absorb the secondary metabolites. The resin was filtered through a cheesecloth and eluted 3 times with 200 mL of acetone. The acetone was evaporated using rotary evaporator. The remaining liquid containing the crude extracts was further extracted 3 times with ethyl acetate in a 1 : 1 ratio (v/v) and the ethyl acetate was evaporated and concentrated with rotary evaporator. The crude extracts containing the secondary metabolites were transferred to 20 mL beakers and covered with foil and stored at room temperature to dry out until further analysis.

#### 2.4.2. Minimum Inhibitory Concentration (MIC)

Minimum inhibitory concentration studies of the bacterial endophyte's crude extracts were performed according to the method by [16] with some modifications. Briefly, stock solutions of the extracts were prepared by dissolving 0.02 g in 1 mL dimethyl sulfoxide (DMSO) to produce a final concentration of 20 mg/mL, which were then serially diluted to concentrations of 10 mg/mL, 5 mg/mL, 2.5 mg/mL, 1.25 mg/mL, 0.625 mg/mL, and 0.312 mg/mL using Mueller-Hinton broth. The pathogenic test strains used were Gram-negative bacteria *Escherichia coli* (ATCC25922), *Pseudomonas aeruginosa* (ATCC27853), and *Klebsiella oxytoca* (ATCC13182) and Gram-positive bacteria *Staphylococcus aureus* (NCTC6571) and *Bacillus cereus* (ATCC10876). Using McFarland 0.5 standard, 50 *μ*L of each pathogen was inoculated in 15 mL of Muller-Hinton broth and incubated at 37°C for 24 hours. Following aseptic techniques in 96 well microtitre plates, 100 *μ*L of pathogenic test strains was added horizontally and 100 *μ*L of the diluted crude extract concentrations was added vertically starting from high to low concentrations. Negative controls [100 *μ*L DMSO and 100 *μ*L sterile Mueller-Hinton broth, 1 : 1 ratio (v/v)] and positive control 1 mg/mL Streptomycin antibiotic (Sigma-Aldrich, Switzerland) were added vertically in the wells subsequent to those with low concentrations. The microtitre plates were incubated at 37°C for 24 hours. The MIC assays were conducted in triplicates. After incubation, ten microliters of resazurin sodium salt solution [0.02% (w/v)] was added to the wells as an indicator of microbial growth and incubated for another 2 hours. The ability of the crude extracts to inhibit pathogenic strains was indicated by no colour change of resazurin sodium salt solution (remains blue) and the colour change from blue to pink after incubation indicated no bacterial inhibition. The MIC (lowest concentration of each extract displaying no visible growth) values were visually determined.

### 2.5. Metabolite Fingerprinting Analysis Using GC-HTOF-MS

Metabolite profiling of the crude extracts was performed at a two-dimensional mode using Pegasus GC-HRTOF-MS system (Leco Corporation St. Joseph, MI, USA). One microliter of each sample was injected and helium was used as the carrier gas at 1 mL/min flow rate. The primary column was a Restek Rtx-5siLMS (30 m × 250 *μ*m d.f.) and the secondary column was Restek Rxil7siLMS (1 m × 250 *μ*m × 0.25 *μ*m d.f.). The GC oven temperature was maintained at 60°C for 1 minute, then ramped to 330°C at 10°C/min, and thereafter held for 5 minutes at transfer line temperature of 280°C. The inlet temperature was 250°C. The MS was optimized at −70 eV (electron energy) with an ion source at 250°C. The collected mass range was 40 to 660 *m*/*z* with an acquisition rate of 10 spectra/second. The generated data was analysed using ChromaTOF® software. The NCBI Pubchem database, available at https://pubchem.ncbi.nlm.nih.gov/ [17], was used to interpret functional groups and PASS online database, available at http://www.way2drug.com/passonline, was used to predict the biological activities of the metabolites [18].

### 2.6. Statistical Analysis

The MIC of the crude's extracts was statistically validated by one-way analysis of variance (ANOVA) using in Microsoft Excel 2016. Significant differences at *p* ≤ 0.05 were considered statistically different.

## 3. Results and Discussion

### 3.1. Morphological Identification

In this study, a total of 11 bacterial endophytes from 5 genera belonging to two phyla (Firmicutes and Proteobacteria) were isolated from surface sterilized leaves and roots of a healthy medicinal plant *D. anomala*. Majority of the endophytic bacteria were isolated from the roots with 6 isolates, followed by the leaves with 5 isolates (Table 1). A great morphological diversity was observed where each isolate showed unique characteristics in terms of colony colour, shape, size, and margins. The isolates were grouped into two distinct classes based on Gram staining technique, The Gram-positive bacteria (7 isolates) and the Gram-negative bacteria (4 isolates).

The surface sterilization technique was adequate as the control plates had shown no microbial growth. Therefore, bacterial colonies that grew on the media were regarded as endophytic bacteria of *D. anomala*. To the best of our knowledge, this is the first report on isolation of bacterial endophytes from *D. anomala*. *Bacillus* genus occurred in both leaves and root samples, having a larger occurrence with 5 strains. These results are comparable to various studies that have reported different genera of Firmicutes and Proteobacteria which included *Bacillus* [19], *Staphylococcus* [20], *Stenotrophomonas* [21], *Enterobacter* [22], and *Pantoea* [23]; bacterial species from these genera seem to be occurring as bacterial endophyte communities of many plants.

The distribution and diversity of bacterial endophytes in plants are influenced by many factors including host plant species, age and type of the plant tissue, geographical and habitat distribution, sampling season, surface sterilization method, growth media, and culture conditions [24]. In this study, the plant material was collected from a slit soil in April 2018, we utilized nutrient agar for growth and isolation of the bacterial endophytes, and as such this resulted in limited number of identified bacterial endophytes.

### 3.2. Molecular Identification and Phylogenetic Analysis of the Bacterial Endophytes

**Figure 1:**
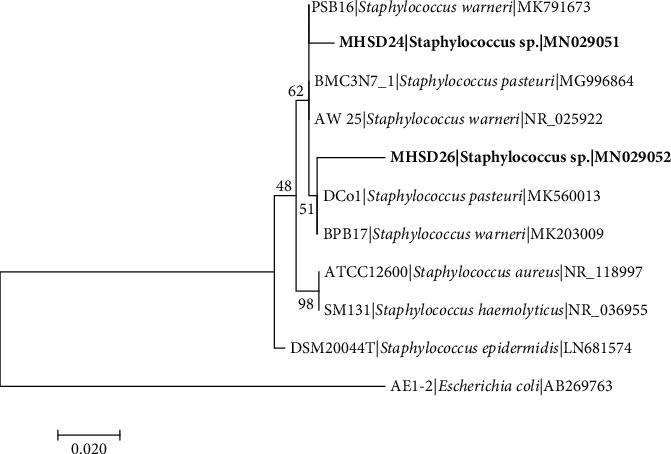
Maximum likelihood phylogenetic tree based on analysis of partial 16S rDNA nucleotide sequences of *Staphylococcus* sp. strain MHSD24 and MHSD26 with related strains from *Staphylococcus* genus. Numbers above or below the nodes indicate bootstrap values generated after 1000 replications. *Escherichia coli* AE-1 (AB269763) was used as an outgroup.

**Figure 2:**
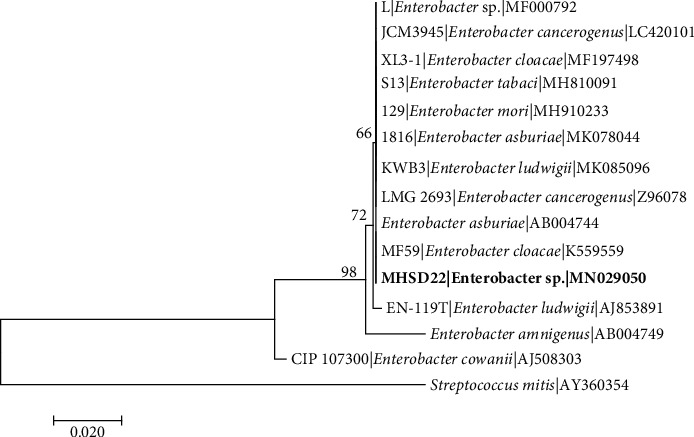
Maximum likelihood phylogenetic tree based on analysis of partial 16S rDNA nucleotide sequences of *Enterobacter* sp. strain MHSD22 with related strains from *Enterobacter* genus. Numbers above or below the nodes indicate bootstrap values generated after 1000 replications. *Streptococcus mitis* (AY360354) was used as an outgroup.

**Figure 3:**
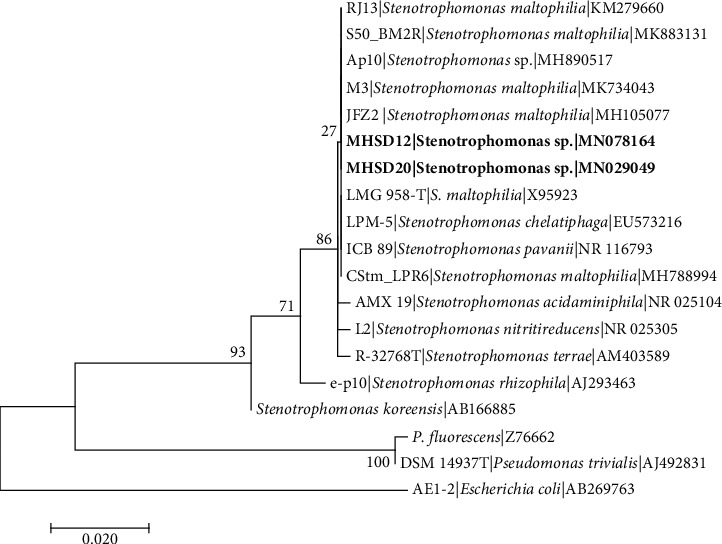
Maximum likelihood phylogenetic tree based on analysis of partial 16S rDNA nucleotide sequences of *Stenotrophomonas* sp. strain MHSD20 with related strains from *Stenotrophomonas* genus. Numbers above or below the nodes indicate bootstrap values generated after 1000 replications. *Escherichia coli* AE-1 (AB269763) was used as an outgroup.

**Figure 4:**
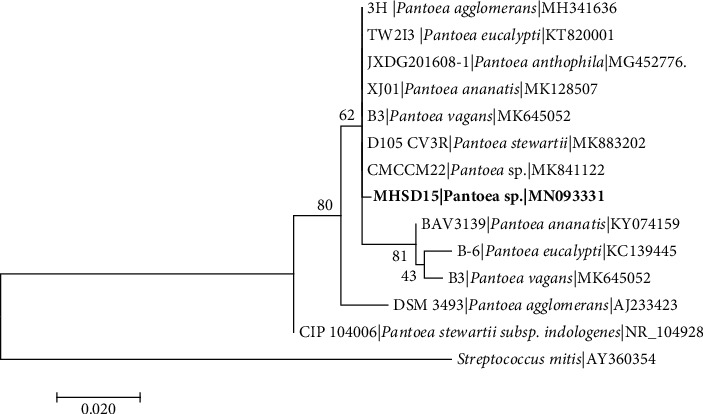
Maximum likelihood phylogenetic tree based on analysis of partial 16S rDNA nucleotide sequences of *Pantoea* sp. strain MHSD15 with related strains from *Pantoea* genus. Numbers above or below the nodes indicate bootstrap values generated after 1000 replications. *Streptococcus mitis* (AY360354) was used as an outgroup.

**Figure 5:**
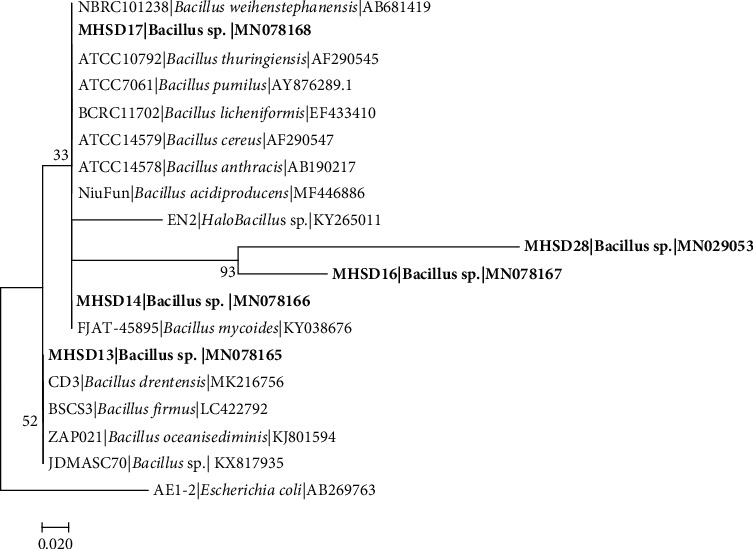
Maximum likelihood phylogenetic tree based on analysis of partial 16S rDNA nucleotide sequences of *Bacillus sp*. strain MHSD28. *Bacillus* sp. strain MHSD13. *Bacillus* sp. strain MHSD14, *Bacillus* sp. strain MHSD16, and *Bacillus* sp. strain MHSD17 with related strains from *Bacillus* genus. Numbers above or below the nodes indicate bootstrap values generated after 1000 replications. *Escherichia coli* AE-1 (AB269763) was used as an outgroup.

The 16S rRNA gene sequence-based identification is a rapid method for molecular identification of bacterial species. Firmicutes and Proteobacteria were found among the isolated endophytic bacteria (Table 2). The bacterial isolates were classified into five genera which include *Bacillus* with 5 five isolates, *Staphylococcus* with 2 isolates, *Stenotrophomonas* with 1 isolate, *Enterobacter* with 1 isolate, and *Pantoea* with 1 isolate, indicating *Bacillus* as the most dominant genus. The 16S rDNA sequences obtained were submitted to NCBI and assigned the accession numbers provided in Table 2. Each genus was analysed in separate phylogenetic trees (Figures 1–5).

*Staphylococcus* sp. strain MHSD24 and MHSD26 sequences were aligned with strains of *Staphylococcus* genus, and *Escherichia coli* AE-1 (AB269763) was used as an outgroup (Figure 1). Strain MHSD24 was closely related to *Staphylococcus warneri* MK791673 with 98.29% similarity whilst strain MHSD26 was closest to *Staphylococcus pasteuri* MK875469 with 97.44% similarity (Table 2). Phylogenetic analysis shows that strain MHSD26 had a polyphyletic cluster with *Staphylococcus pasteuri* DCo1 and *S. warneri* BPB17, and strain MHSD24 also had a polyphyletic relationship with *S. warneri* PSB16. Numbers above or below the nodes indicate bootstrap values generated after 1000 replications.

*Enterobacter* sp. strain MHSD22 was closely related to *Enterobacter ludwigii* KU054383 with 97.48% similarity (Table 2) and phylogenetic analysis showed strain MHSD22 had polyphyletic relationship with closely related *Enterobacter* species; *Streptococcus mitis* (AY360354) was used as an outgroup (Figure 2).

*Stenotrophomonas* strain MHSD20 and MHSD12 were aligned with closely related species of *Stenotrophomonas* genus, and *Escherichia coli* AE-1 (AB269763) was used as an outgroup taxon (Figure 3). Strain MHSD20 was closely related to *Stenotrophomonas maltophilia* (KM279660) with 98.55% similarity and strain MHSD12 was closely related to *Stenotrophomonas maltophilia* (MK734043) with 96.85% similarity (Table 2). Phylogenetically, both strains showed high similarity among themselves, having a polyphyletic relationship with closely related *Stenotrophomonas* species (Figure 3). *Pantoea* sp. strain MHSD15 had a polyphyletic relationship with closely related species (Figure 4) and it had a 92.70% similarity with *Pantoea sp*. (MH769026) (Table 2).

Similarly, the sequences from that of *Bacillus* sp. strain MHSD28 were closely related to *Bacillus cereus (*MK503979) with 97.88% similarity, *Bacillus* sp. strain MHSD13 was closely related to *Bacillus infantis* (MK850860) with 97.33% similarity, *Bacillus* sp. strain MHSD14 was closely related to *Bacillus thuringiensis* (LT838181) with 97.68% similarity, *Bacillus* sp. strain MHSD16 was closely related to *Bacillus s*p. (EUC01244) with 90.96% similarity, and *Bacillus* sp. strain MHSD17 was closely related to *Bacillus cereus* (KJ935725) with 98.15% similarity as indicated in Table 2. Phylogenetic analysis showed sister relationship between strain MHSD28 and MHSD16, strain MHSD17 and MHSD14 formed a polyphyletic relationship with related *Bacillus* species, and MHSD13 also had a polyphyletic relationship with closely related species (Figure 5).

The isolated bacterial endophytes were identified to genus level using the 16S rRNA gene phylogenetic analysis; we suggest sequencing other genes other than the 16S rRNA for further species description and phylogenetic delineation. The 16S rRNA gene has been recommended for identification of bacteria and this technique is regarded as the gold standard for species classification because the gene is ubiquitous in prokaryotes, containing approximately 1500 bases which are adequate for species analysis [25]. The 16S RNA genes also consist of variable regions that enable comparison of species between distantly related species and 16S rRNA gene is less likely to undergo horizontal gene transfer (HGT) [42]. Although the 16S rRNA gene is suitable for bacterial identification [26], many bacterial species cannot be differentiated by their 16S rRNA gene sequences as it gives high similarity between closely related species which is also indicated by the formation of polyphyletic relationships on a phylogenetic tree [25]. Bacterial endophytes identified in this study formed polyphyletic relationship with closely related strains. In a separate study [27], based on phylogenomic analysis, it was indicated that *Stenotrophomonas* strain MHSD12 is closely related to *Stenotrophomonas pavanii* strain DSM 25135 which is an endophyte isolated from sugarcane [28].

### 3.3. Antibacterial Assay Using Minimum Inhibition Concentration

The antibacterial activity of endophytic bacteria's crude extracts was determined against five pathogenic test strains: Gram-negative bacteria *Escherichia coli* (ATCC25922), *Pseudomonas aeruginosa* (ATCC27853), and *Klebsiella oxytoca* (ATCC13182); Gram-positive bacteria *Staphylococcus aureus* (NCTC6571) and *Bacillus cereus* (ATCC10876). The MIC activity varied depending on the test strain used, most extracts showed interesting results, and the positive control Streptomycin at 1 mg/mL was effective against all the test strains. Minimum inhibition concentration of the extracted crude extracts from bacterial endophyte ranged from 0.312 mg/mL to 10 mg/mL. The lowest MIC value was 0.312 mg/mL and other crude extracts indicated MIC values higher than 1 mg/mL as shown in Table 3.

Bacterial endophyte *Stenotrophomonas* sp. strain MHSD20 had MIC values ranging from 0.625 mg/mL to 5 mg/mL, inhibiting *B. cereus* at 0.625 mg/mL. *Enterobacter* sp. strain MHSD22 showed MIC values from 1.25 mg/mL to 10 mg/mL which were considered noninhibiting. The crude extract of *Staphylococcus* sp. strain MHSD26 had MIC concentration values ranging from 0.312 mg/mL to 10 mg/mL, inhibiting *B. cereus* and *S. aureus* at 0.625 and 0.312 mg/mL, respectively. *Bacillus* sp. strain MHSD28 showed MIC values ranging from 0.312 mg/mL to 10 mg/mL, inhibiting *S. aureus* 0.312 mg/mL and *E. coli* at 0.625 mg/mL. Crude extracts of *Bacillus* sp. strain MHSD14 had MIC values of 0.312 mg/mL to 5 mg/mL inhibiting *S. aureus* at 0.312 mg/mL and finally *Pantoea* sp. strain MHSD15 crude extracts had MIC values of 0.625 mg/mL to 10 mg/mL inhibiting *S. aureus* at 0.625 mg/mL. These findings are congruent with several studies, which revealed that endophytic bacteria have proven to be potential reliable sources of novel bioactive compounds with antimicrobial activity [10]. The MIC values of crude extracts <1 mg/mL are classified to have significant antibacterial activity while extracts with MIC values > 1 mg/mL are classified as noninhibitors [29]. Most of the endophyte's crude extracts had significant inhibition values with the lowest MIC values 0.625 mg/mL and 0.312 mg/mL. *Enterobacter* sp. strain MHSD22 did not have significant inhibition against all the test strains. The MIC values of the crude extracts at 0.625–0.325 mg/mL were significantly different (*p* < 0.05), with the exception of *Enterobacter* sp. strain MHSD22 which had MIC values higher than 1 against all test strains.

The difference in the inhibition between Gram-positive and Gram-negative may be due to variation in their cell wall structure. Gram-negative bacteria have an extra outer membrane, which could increase impermeability to antimicrobials. Similar results were observed in other studies [30]. The antibacterial activities of crude extracts of the bacterial endophytes obtained in this study provide evidence that endophytic bacteria could have potential in outcompeting pathogenic bacteria and this necessitates their use in drug discovery.

### 3.4. Gas Chromatography High-Resolution Time-of-Flight Mass Spectrometry Analysis

Bacterial endophytes are reported to produce same or similar bioactive compounds produced by the host plant [1], thus making them a promising source of novel molecules with various biotechnological applications. *Dicoma anomala* has ethnomedicinal history for the treatment of coughs, cold fever, and labour pains with pharmacological potential to antibacterial, anti-inflammatory, and antiplasmodial activities that could be a potential target for bioactive secondary metabolites studies [8]. Different classes of secondary metabolites such as phenolic acids, flavonoids, and triterpenes had previously been identified from *D. anomala* crude extracts [8]. In the current study, we identified 15 compounds from ethyl acetate crude extracts of bacterial endophytes isolated from *D. anomala*. Identification and characterization of secondary metabolites can lead to discovery of new compounds for drug development [31] including discovery of compounds that are of interest in food, cosmetic, and other industries. Most of the identified compounds in this study have been reported to possess interesting biological activities ranging from antimicrobial, antioxidant, and anti-inflammatory as summarized in Table 4. Benzyl benzoate is reported to function as fragrance ingredient, pesticides, pH adjusters, preservatives, solvents, and/or viscosity decreasing agents in cosmetic products [32]. Benzyl benzoate was identified in both *Enterobacter* sp. strain MHSD22 and *Stenotrophomonas* sp. strain MHSD12 bacterial endophytes extracts and it is reported to have antibacterial activity [33]. The identification of this compound indicates that, in addition to discovery of drug developments, bacterial endophytes are also useful for discovery of other bioactive compounds beneficial in food, cosmetic, and chemical industry [34]; this discovery necessitates further studies.

9-Octadecenamide, (Z)- is an oleamide, an amide derived oleic acid biosynthesis that has shown antioxidative and hypolipidemic bioactivities [35]. It was previously identified from *Bacillus* crude extracts isolated from the rhizosphere of groundnut [36]. According to Cheng et al. [35], this compound was also reported as a potential medicinal treatment for mood and sleeping disorders; it was identified in *Stenotrophomonas* sp. strain MHSD12 and *Pantoea* sp. strain MHSD15 bacterial endophytes extracts. Hexadecane was identified in all bacterial endophyte extracts with *Stenotrophomonas* sp. strain MHSD20 as an exception. It is known to possess antifungal and antibacterial as well as antioxidant activity [35, 37]. Pyrrolo[1,2-a] pyrazine-1,4-dione, hexahydro-3-(2-methylpropyl)- was identified in *Staphylococcus* sp. strain MHSD26, *Enterobacter* sp. strain MHSD22, and *Bacillus* sp. strain MHSD28 extracts and this compound has been reported as an antibiotic, anti-inflammatory, cholesterol reducing, and antitumor drug agent [38].

Heptacosane has been reported to have antioxidant properties [39] and was previously identified from *Pseudomonas* spp. isolated from *Vigna radiate* [40]. In the current study, heptacosane was identified from *Bacillus* sp. strain MHSD28, *Bacillus* sp. strain MHSD14, *Stenotrophomonas* sp. strain MHSD12, *Staphylococcus* sp. strain MHSD26, and *Pantoea* sp. strain MHSD15 extracts. Most of the compounds identified in this study have antimicrobial activity, which explains the significant antibacterial activity of the crude extracts. To our knowledge, this is the first study to report for the first time bacterial endophytes associated with *D. anomala*, with antimicrobial activity against bacterial pathogens and non-targeted profiling of their secondary metabolites.

## 4. Conclusions

Based on the results, we conclude that *D. anomala* does harbor diverse types of endophytic bacteria. Moreover, antimicrobial activity of these endophytes' crude extracts was significant against pathogenic strains. Further investigation should be conducted to isolate biologically active compounds from bacterial endophytes with antimicrobial activity for drug development and use in other industries such as food and cosmetics.

## Figures and Tables

**Table 1:** Morphological characteristics of bacterial endophytes isolated from leaves and roots tissues of *Dicoma anomala*.

Plant part	Bacterial sample code	Assigned isolate name	Phyla	^*∗*^Gram reaction	Cell shape
Leaves	SC10L	*Stenotrophomonas* sp. strain MHSD20	Proteobacteria	−ve	Rods
	SC7L	*Enterobacter* sp. strain MHSD22	Proteobacteria	−ve	Rods
	MS3L	*Staphylococcus* sp. strain MHSD24	Firmicutes	+ve	Cocci
	MS4L	*Staphylococcus* sp. strain MHSD26	Firmicutes	+ve	Cocci
	MS8L	*Bacillus* sp. strain MHSD28	Firmicutes	+ve	Rods
Roots	SC4R	*Stenotrophomonas* sp. strain MHSD12	Proteobacteria	−ve	Rods
	SC1R	*Bacillus* sp. strain MHSD13	Firmicutes	+ve	Rods
	SC2R	*Bacillus* sp. strain MHSD14	Firmicutes	+ve	Rods
	SC5R	*Pantoea* sp. strain MHSD15	Proteobacteria	−ve	Rods
	MS3R	*Bacillus* sp. strain MHSD16	Firmicutes	+ve	Rods
	MS10 R	*Bacillus* sp. strain MHSD17	Firmicutes	+ve	Rods

^*∗*^Gram reaction: −ve = Gram-negative; +ve = Gram-positive.

**Table 2:** NCBI BLAST 16S rRNA gene sequences of bacterial endophytes isolated from *Dicoma anomala*.

Assigned bacterial name	Assigned GenBank accession number	NCBI blast results			
		Closest related species with accession number	Query coverage %	*E*-value	Identity similarity %
*Stenotrophomonas* sp. strain MHSD20	MN029049	*Stenotrophomonas maltophilia* KM279660	100	0.0	98.55
*Enterobacter* sp. strain MHSD22	MN029050	*Enterobacter ludwigii* KU054383	100	0.0	97.48
*Staphylococcus* sp. strain MHSD24	MN029051	*Staphylococcus warneri* MK791673	100	0.0	98.29
*Staphylococcus* sp. strain MHSD26	MN029052	*Staphylococcus pasteuri* MK875469	100	0.0	97.44
*Bacillus* sp. strain MHSD28	MN029053	*Bacillus cereus* MK503979	100	0.0	97.88
*Stenotrophomonas* sp. strain MHSD12	MN078164	*Stenotrophomonas maltophilia* MK734043	100	0.0	96.85
*Bacillus* sp. strain MHSD13	MN078165	*Bacillus infantis* MK850860	100	0.0	97.33
*Bacillus* sp. strain MHSD14	MN078166	*Bacillus thuringiensis* LT838181	100	0.0	97.68
*Pantoea* sp. strain MHSD15	MN093331	*Pantoea sp*. MH769026	100	0.0	92.70
*Bacillus* sp. strain MHSD16	MN078167	*Bacillus s*p. EUC01244	99	0.0	90.96
*Bacillus* sp. strain MHSD17	MN078168	*Bacillus cereus* KJ935725	99	0.0	98.15 %

**Table 3:** Minimum inhibitory concentrations of crude extracts of secondary metabolites from bacterial endophytes associated with *Dicoma anomala.*

	Pathogenic test strains				
	*E. coli* (ATCC25922)	*B. cereus (*ATCC10876)	*S. aureus* (NCTC6571)	*P. aeruginosa* (ATCC27853)	*K. oxytoca* (ATCC13182)
Bacterial Endophyte's crude extracts	MIC (mg/mL)				
*Stenotrophomonas* sp. strain MHSD20	2.5	0.625	2.5	1.25	5
*Enterobacter* sp. strain MHSD22	2.5	5	2.5	5	10
*Staphylococcus* sp. strain MHSD26	5	0.625	0.312	2.5	10
*Bacillus* sp. strain MHSD28	0.625	2.5	0.312	2.5	2.5
*Stenotrophomonas* sp. strain MHSD12	1.25	0.625	2.5	0.625	5
*Bacillus* sp. strain MHSD14	5	0.312	1.25	5	2.5
*Pantoea* sp. strain MHSD15	1.25	2.5	0.625	2.5	10

^*∗*^Streptomycin (positive control) inhibited all the test strains at 1 mg/mL.

**Table 4:** GC-HRTOFMS analysis bacterial endophyte's crude extracts associated with *Dicoma anomala*.

RT_(min)_	*m*/*z*	Area%	Molecular formular	Name of the compound	Biological activity	Bacterial endophyte	References
15.51	212.0833	0.08	C_14_H_12_O_2_	Benzyl benzoate	Fragrance ingredients, pesticides, pH adjusters, preservatives, solvents, and/or viscosity decreasing agents in cosmetic products	E5, E2,	[32]
14.00	491.0536	0.32	C_16_H_48_O_8_Si_8_	Cyclohexasiloxane, dodecamethyl-	Preservative	E5	[41]
21.46	281.2712	0.11	C_19_H_36_O_2_	9-Octadecenamide, (Z)-	Antioxidative and hypolipidemic bioactivity	E5, E7	[35]
17.65	226.0927	0.89	C_16_H_34_	Hexadecane	Antifungal, aAntibacterial, antioxidant activity	E4, E6, E5, E2, E3, E7	[35, 37]
17.38	208.1223	2.38	C_11_H_18_N_2_O_2_	Pyrrolo[1,2-a] pyrazine-1,4-dione, hexahydro-3-(2-methylpropyl)-	Antibiotics, anti-inflammatory drugs, cholesterol reducing drugs and antitumor agents	E3, E2, E4	[38]
17.76	549.0229	0.14	C_16_H_5_0O_7_Si_8_	Octasiloxane, 1, 1, 3, 3, 5, 5, 7, 7, 9, 9, 11, 11, 13, 13, 15, 15-hexadecamethyl-	Antimicrobial	E5, E2	[38]
28.48	532.9910	0.19	C_16_H_48_O_6_Si_7_	Heptasiloxane, hexadecamethyl-	Nematicide, antiantrogenic, anticoranary, and antieczemic	E5, E2, E7, E1	[42]
19.57	236.0503	0.00	C_14_H_18_O_4_	Phthalic acid, methyl 2-pentyl ester	Plasticizers, phenol derivatives used for flexibility and durability of plastic used in cosmetics, perfumes, food packaging, toys and medical devices, antineoplastic and immunosuppressive	E5,	[43, 44]
25.80	269.2484	0.09	C_16_H_22_O_4_	Dibutyl phthalate	Antifungal, antibacterial, antiviral, and antioxidant activities	E4, E6, E5, E2	[45]
8.33	117.0574	0.32	C_8_H_7_N	Indole	Anti-inflammatory and analgesic agents	E4, E6, E5, E2, E3, E7	[46]
18.00	283.3320	0.69	C_27_H_56_	Heptacosane	Antioxidant activity	E7, E3, E5, E6, E4	[39]
17.98	280.3130	0.03	C_20_H_40_	9-Eicosene	Antimicrobial and cytotoxic properties	E3, E5, E6	[47]
11.97	194.0939	0.04	C_11_H_14_O_3_	Benzoic acid, 4-ethoxy-, ethyl ester	Antimicrobial preservative	E5, E7, E6	[48]
19.44	287.9506	0.03	C_12_H_7_Cl_3_O_2_	Triclosan	Antimicrobial	E6, E2, E3, E7	[49]
6.21	99.0316	0.24	C_4_H_5_NO_2_	Succinimide	CNS depressant, analgesic, antitumor, cytostatic, anorectic, nerve conduction blocking, antispasmodic, bacteriostatic, muscle relaxant, hypotensive, antibacterial, antifungal, anticonvulsant and antitubercular	E7	[50]

RT (m) = retention time (minutes), *m*/*z* = mass-to-charge ratio, E1∗(*Stenotrophomonas* sp. strain MHSD20), E2∗(*Enterobacter* sp. strain MHSD22), E3∗(*Staphylococcus* sp. strain MHSD26), E4∗(*Bacillus* sp. strain MHSD28), E5∗(*Stenotrophomonas* sp. strain MHSD12), E6∗(*Bacillus* sp. strain MHSD14), E7∗(*Pantoea* sp. strain MHSD15).

## Data Availability

The 16S rRNA gene data of this study are available from the corresponding author upon request. The reported bacterial endophytes in this study have been deposited in GenBank with the following accession numbers: MN029049, MN029050, MN029051, MN029052, MN029053, MN078164, MN078165, MN078166, MN093331, MN078167, and MN078168.
